# Genome Sequence of *Aeromonas taiwanensis* LMG 24683^T^, a Clinical Wound Isolate from Taiwan

**DOI:** 10.1128/genomeA.00579-14

**Published:** 2014-06-12

**Authors:** Hsuan-Chen Wang, Wen-Chien Ko, Hung-Yu Shu, Po-Lin Chen, Yu-Chun Wang, Chi-Jung Wu

**Affiliations:** a National Institute of Infectious Diseases and Vaccinology, National Health Research Institutes, Tainan, Taiwan; b Department of Internal Medicine, National Cheng Kung University, College of Medicine and Hospital, Tainan, Taiwan; c Department of Bioscience Technology, Chang Jung Christian University, Tainan, Taiwan

## Abstract

*Aeromonas taiwanensis* was first described in 2010 on the basis of one clinical wound isolate (strain LMG 24683^T^ = A2-50^T^ = CECT 7403^T^) from Taiwan. We present here the genome sequence of *A. taiwanensis* LMG 24683^T^, which carries several genes encoding virulence determinants and Ambler class C and D β-lactamases.

## GENOME ANNOUNCEMENT

Members of the genus *Aeromonas* are found in aquatic environments worldwide and have been implicated in human diseases. *Aeromonas taiwanensis* was first described in 2010 on the basis of one strain (A2-50^T^ = LMG 24683^T^ = CECT 7403^T^) recovered from the wound of a hospitalized Taiwanese patient (1). An unrooted phylogenetic tree derived from a multilocus phylogenetic analysis of five housekeeping genes allocated the strain to a novel species, and *Aeromonas caviae* was found to be a neighboring species (1). So far, one additional clinical fecal and four environmental *A. taiwanensis* isolates have been recorded (2, 3). Considering the clinical relevance of *A. taiwanensis*, the genome sequence was analyzed to facilitate further studies on its virulence and antimicrobial resistance.

Whole-genome sequencing of *A. taiwanensis* LMG 24683^T^ was performed using the 454 sequencing technology. Genomic shotgun and 8-kb paired-end libraries were constructed and sequenced according to the instruction for the 454-GS Junior instrument (Roche Diagnostics, Indianapolis, IN). A total of 98,851,428 bp in 228,474 reads from the shotgun library and 73,608,666 bp in 172,799 reads from the 8-kb paired-end library were assembled into 104 contigs using 454 Newbler (version 2.7; 454 Life Sciences, Branford, CT). Using the connecting paired-end reads, these contigs were clustered into 14 scaffolds. The final assembly of the genome sequences of LMG 24683^T^ contains a circular chromosome of 4,230,588 bp. The deduced genome size of LMG 24683^T^ is 4.35 Mb (the scaffold size is 4,347,025 bp), which is smaller than that of *Aeromonas dhakensis* AAK1 (formerly *Aeromonas aquariorum*; 4.81 Mb) (4), *Aeromonas hydrophila* ATCC 7966^T^ (4.74 Mb) (5), *Aeromonas veronii* B565 (4.55 Mb) (6), and *A. caviae* Ae398 (4.43 Mb) (7). Pairwise genome alignments using 454 Newbler showed that the LMG 24683^T^ genome displays the highest overall synteny with *A. caviae* Ae398 (69.7% aligned), followed by *A. dhakensis* AAK1 (60.0% aligned), *A. hydrophila* ATCC 7966 (58.1% aligned), and *A. veronii* B565 (43.5% aligned).

The genome sequence was annotated using CLC Genomics Workbench 5.5.1 (CLC bio, Aarhus, Denmark) and the NCBI Basic Local Alignment Search Tool. Similar to previous PCR results from two *A. taiwanensis* strains (10A4A and 4A7A) (2), LMG 24683^T^ was positive for the genes that encode hemolysin, flagella (*fla*), lateral flagella (*lafA*), elastase (*ahpB*), and lipase (*pla/lipH3/apl-l/lip*) but negative for those that encode cytotonic enterotoxin (*ast*) and cytotoxic enterotoxin (*act*). Additionally, LMG 24683^T^ was found to carry a gene that has 85% sequence identity to the *A. hydrophila* cytotonic enterotoxin gene (*alt*) (GenBank accession no. DQ302131). Other putative virulence determinants found include genes that encode type IV pilus, type IV fimbriae, toxin transporter, metalloprotease, extracellular protease, phospholipase A1, hyaluronidase, siderophore synthesis, ferric uptake regulator, enolase, DNA adenine methyltransferase, glucose-inhibited division protein, autoinducer synthase, and ribosylhomocysteine lyase. LMG 24683^T^ also possesses the genes encoding Ambler class C and D β-lactamases but not the gene encoding class B CphA-related metallo-β-lactamase, a profile similar to that noted for *A. caviae*. Although the functionality of these genes needs to be validated, knowledge of the genome sequence of *A. taiwanensis* opens new avenues for further exploring important virulence determinants.

### Nucleotide sequence accession numbers. 

This assembly was deposited at the WGS division of DDBJ/EMBL/GenBank under accession no. BAWK01000001 to BAWK01000104. The version described in this paper is the first version.
